# Parkinson's disease-linked mutations in *VPS35* induce dopaminergic neurodegeneration

**DOI:** 10.1093/hmg/ddu178

**Published:** 2014-04-15

**Authors:** Elpida Tsika, Liliane Glauser, Roger Moser, Aris Fiser, Guillaume Daniel, Una-Marie Sheerin, Andrew Lees, Juan C. Troncoso, Patrick A. Lewis, Rina Bandopadhyay, Bernard L. Schneider, Darren J. Moore

**Affiliations:** 1 Laboratory of Molecular Neurodegenerative Research; 2 Neurodegenerative Disease Laboratory, Brain Mind Institute, School of Life Sciences, Ecole Polytechnique Fédérale de Lausanne (EPFL), 1015 Lausanne, Switzerland; 3 Department of Molecular Neuroscience; 4 Queen Square Brain Bank for Neurological Disorders, University College London Institute of Neurology, London WC1N 3BG, UK; 5 Department of Pathology, Johns Hopkins University School of Medicine, Baltimore, MD 21205, USA; 6 School of Pharmacy, University of Reading, Reading RG6 6AP, UK,; 7 Reta Lila Weston Institute of Neurological Studies, University College London Institute of Neurology, London WC1N 1PJ, UK; 8 Center for Neurodegenerative Science, Van Andel Institute, Grand Rapids, MI 49503, USA

## Abstract

Mutations in the *vacuolar protein sorting 35 homolog* (*VPS35*) gene at the *PARK17* locus, encoding a key component of the retromer complex, were recently identified as a new cause of late-onset, autosomal dominant Parkinson's disease (PD). Here we explore the pathogenic consequences of PD-associated mutations in VPS35 using a number of model systems. VPS35 exhibits a broad neuronal distribution throughout the rodent brain, including within the nigrostriatal dopaminergic pathway. In the human brain, VPS35 protein levels and distribution are similar in tissues from control and PD subjects, and VPS35 is not associated with Lewy body pathology. The common D620N missense mutation in VPS35 does not compromise its protein stability or localization to endosomal and lysosomal vesicles, or the vesicular sorting of the retromer cargo, sortilin, SorLA and cation-independent mannose 6-phosphate receptor, in rodent primary neurons or patient-derived human fibroblasts. In yeast we show that PD-linked VPS35 mutations are functional and can normally complement *VPS35* null phenotypes suggesting that they do not result in a loss-of-function. In rat primary cortical cultures the overexpression of human VPS35 induces neuronal cell death and increases neuronal vulnerability to PD-relevant cellular stress. In a novel viral-mediated gene transfer rat model, the expression of D620N VPS35 induces the marked degeneration of substantia nigra dopaminergic neurons and axonal pathology, a cardinal pathological hallmark of PD. Collectively, these studies establish that dominant *VPS35* mutations lead to neurodegeneration in PD consistent with a gain-of-function mechanism, and support a key role for VPS35 in the development of PD.

## INTRODUCTION

Parkinson's disease (PD) is a common progressive neurodegenerative movement disorder (1,2). The motor deficits of PD result from the relatively selective degeneration of dopaminergic neurons of the substantia nigra pars compacta. PD is characterized neuropathologically by the appearance of Lewy bodies in surviving dopaminergic neurons that are enriched for fibrillar α-synuclein (3). While the clinical and pathologic features of PD are well-defined, the underlying cause of the disease remains enigmatic. In 5–10% of cases, PD is inherited in a familial manner and disease-causing mutations have been identified in at least eight genes (4,5).

Mutations in the *VPS35* gene cause late-onset, autosomal dominant familial PD (6,7). A single missense mutation, Asp620Asn (D620N), was originally shown to segregate with PD in Swiss and Austrian families, and has been identified in a number of PD subjects and families worldwide (6–8). Additional rare *VPS35* variants (i.e. P316S, R524W, I560T, H599R and M607V) may also be linked to PD although their pathogenicity remains unclear. *VPS35* mutations are the second most common cause of late-onset familial PD after *LRRK2* mutations (9). The neuropathological features of *VPS35*-linked PD are not yet known since no mutation carriers have so far come to autopsy although clinical and neuroimaging data suggest a classical disease spectrum similar to idiopathic PD (6,7). The mechanism by which dominantly inherited mutations in *VPS35* precipitate PD is not known.

Human *VPS35* encodes a 796 amino acid protein that forms a horseshoe-shaped α-helical solenoid (10,11). VPS35 is a key subunit of the retromer complex involved in the retrieval and sorting of transmembrane proteins from endosomes to the *trans*-Golgi network (11,12). VPS35 interacts with VPS26A and VPS29 to form a trimeric cargo recognition subcomplex that selectively recognizes and binds to transmembrane cargo proteins (13). This trimeric subcomplex associates with a sorting nexin dimer required for its recruitment to endosomes (13). Retromer substrates include intracellular receptors, such as the cation-independent mannose 6-phosphate receptor (CI-M6PR), sortilin and SorLA (11). The D620 residue of VPS35 is highly conserved from yeast to humans suggesting a potentially important function (6). It is conceivable that the familial D620N mutation may disrupt the proper retromer-dependent trafficking of cargo proteins.

Aside from human genetic data (9), there are limited studies to date exploring a role for VPS35 in PD. Here, we comprehensively explore the pathogenic effects of PD-linked *VPS35* mutations by exploiting numerous model systems, including *Saccharomyces cerevisiae*, patient-derived fibroblasts, primary neuronal cultures and viral-mediated gene transfer in rodents. Our data demonstrate that PD-linked *VPS35* mutations induce neuronal degeneration most likely through a gain-of-function mechanism and provide support for an important contribution of VPS35 to the development of PD.

## RESULTS

### Distribution and levels of VPS35 in the normal and pathological mammalian brain

**Figure 1.**
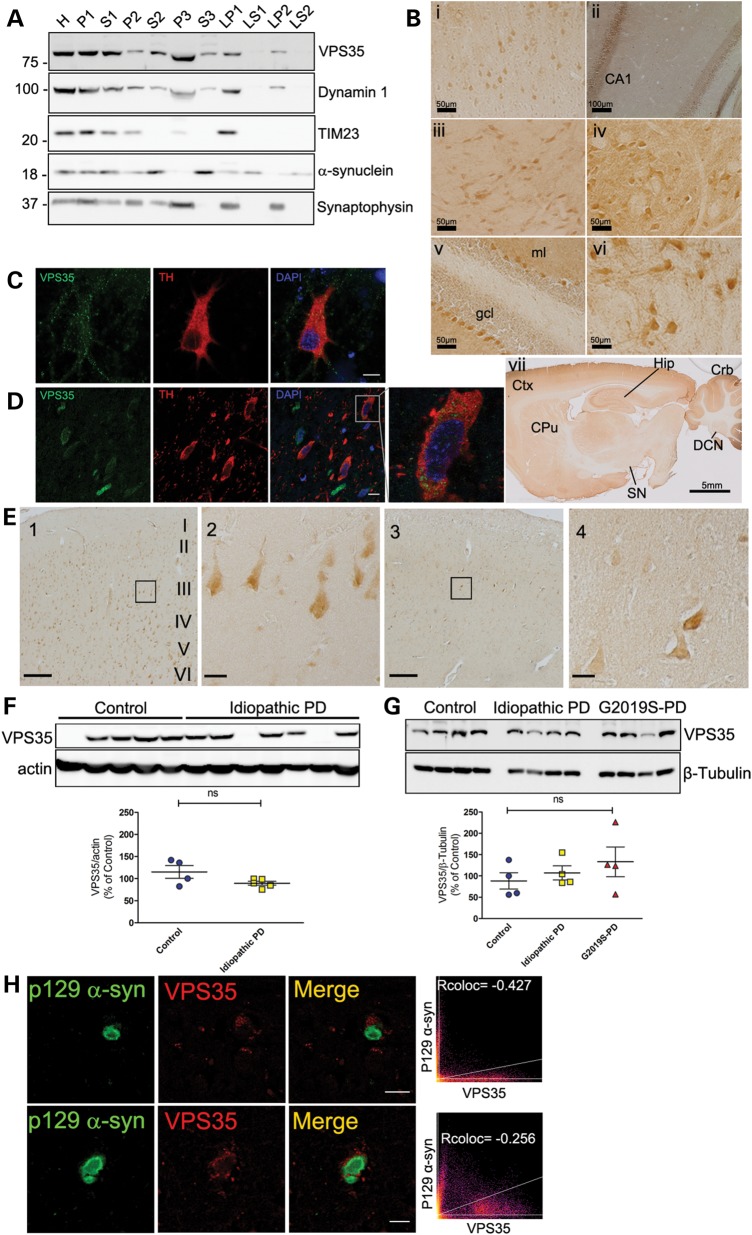
Cellular distribution and levels of endogenous VPS35 in normal and pathological mammalian brain. (**A**) Subcellular fractionation of endogenous VPS35 in mouse cerebral cortex. VPS35 is enriched in the microsomal (P3), synaptosomal (LP1) and synaptic vesicle (LP2) membrane fractions. Dynamin 1, TIM23, α-synuclein and synaptophysin serve as markers for microsomes, mitochondria, synaptic vesicle cytosolic and synaptosomal/synaptic vesicle membranes, respectively. Molecular mass is indicated in kDa. (**B**) Immunolabeling of endogenous VPS35 in the rat brain. VPS35 is detected in (i) pyramidal neurons of cortical layer III, (ii) pyramidal neurons of the hippocampus (CA1 region), (iii) ventral midbrain, (iv) brainstem (superior olivary complex), (v) Purkinje neurons in the cerebellum (granule cell layer, gcl; molecular layer, ml), (vi) deep cerebellar nuclei, and (vii) a sagittal section of rat brain (cerebral cortex, Ctx; hippocampal formation, Hip; cerebellum, Crb; deep cerebellar nuclei, DCN; caudate putamen, CPu; substantia nigra, SN. (**C**) Confocal microscopy analysis of rat primary midbrain cultures immunolabeled with VPS35 and the dopaminergic marker, tyrosine hydroxylase (TH). Nuclei are labeled with DAPI. VPS35 localizes to punctate intracellular vesicular structures within the soma and neuritic processes of TH-positive dopaminergic neurons. Scale bar: 10 μm. (**D**) Co-localization of endogenous VPS35 with TH-positive dopaminergic neurons in the substantia nigra pars compacta of adult rats. Scale bar: 10 μm. (**E**) Immunolabeling of endogenous VPS35 in the human cingulate cortex of control (1) and PD/DLB (3) subjects. Scale bar: 200 μm. High-magnification images of pyramidal neurons from cortical layer III are shown corresponding to the boxed area from control (2) and PD/DLB (4) brains. Scale bar: 50 μm. (**F–G**) Western blot analysis of soluble extracts from ***F*** human caudate putamen of control and idiopathic PD/DLB subjects, and ***G*** human frontal cortex of control, idiopathic PD (PD) or G2019S LRRK2-linked PD subjects, with antibodies to VPS35, and actin or β-tubulin as protein loading controls. Densitometric analysis of VPS35 normalized to actin or β-tubulin levels for individual subjects are shown expressed as a percent of the mean of control subjects. Horizontal bars represent mean ± SEM (*n* = 4–5 subjects/group) for each subject group. Subjects without VPS35 expression in **F** were excluded from the analysis (control = 4 from 5; PD = 5 from 7). ns, non-significant by one-way analysis of variance (ANOVA) with Dunnett's *post hoc* test. (**H**) Confocal microscopic analysis of VPS35 co-localization with Lewy bodies labeled with phospho-Ser129-α-synuclein in cortical layer III neurons from a PD/DLB subject. Correlation coefficients (Rcoloc) and cytofluorograms indicate a lack of co-localization of VPS35 with Lewy bodies. Scale bars: 20 μm (top panels) or 5 μm (bottom panels).

To begin to understand how familial *VPS35* mutations precipitate neurodegeneration in PD, we investigated the normal distribution of endogenous VPS35 in the mammalian brain. Subcellular fractionation of mouse cerebral cortex reveals an enrichment of VPS35 in microsomal vesicles (P3) and at lower levels in crude synaptosomes (LP1) and synaptic vesicle membranes (LP2) (Fig. 1A). Within the rat brain, VPS35 is broadly distributed to multiple neuronal populations including those within the cerebral cortex, hippocampal formation, ventral midbrain, brainstem and cerebellum (Fig. 1B). VPS35 is not particularly enriched within neurons of the nigrostriatal dopaminergic pathway, which selectively degenerate in PD (Fig. 1B). However, confocal microscopic analyses reveal localization of VPS35 to intracellular punctate structures within dopaminergic neurons from rat primary midbrain cultures (Fig. 1C) or the intact rat substantia nigra (Fig. 1D), consistent with the localization of VPS35 to multiple vesicular compartments. Collectively, VPS35 is selectively localized to neuronal vesicular compartments throughout the rodent brain, including substantia nigra dopaminergic neurons that selectively degenerate in PD.

To explore the relationship of VPS35 with PD, the distribution and protein levels of VPS35 were assessed in postmortem human brain tissue from normal control and PD/dementia with Lewy bodies (DLB) subjects (refer to Table 1). VPS35 localizes equivalently to pyramidal neurons throughout all layers of the cingulate cortex of control and idiopathic PD/DLB subjects (Fig. 1E). The steady-state levels of VPS35 are not significantly different in caudate putamen extracts from a series of control and idiopathic PD/DLB subjects (Fig. 1F, refer to Table 1) or in frontal cortex extracts from an independent series of control, idiopathic PD or G2019S LRRK2-linked PD subjects (Fig. 1G, refer to Table 2). VPS35 fails to co-localize with α-synuclein-positive LB pathology within neurons located throughout the cingulate cortex of idiopathic PD/DLB cases but localizes normally to intracellular vesicles within LB-positive and LB-negative neurons from control and PD/DLB brains (Fig. 1H). Our data demonstrate that the neuronal distribution and levels of VPS35 protein are not altered in the brains of PD or DLB subjects. PD-associated mutations in the dominant gene product, *LRRK2* (G2019S), do not alter VPS35 levels within the human brain.

**Table 1.** Clinical details of human brain tissue from JHMI

Subject	Case #	Gender	Age (years)	PMD (h)	Region	Use
Control	1683	F	91	8	CING	IHC
Control	1881	F	48	12	CING	IHC
Control	993	M	66	12	PUT	WB
Control	1361	F	49	15	PUT	WB
Control	1613	M	74	4	PUT	WB
Control	1683	F	91	8	PUT	WB
Control	2052	M	79	16	PUT	WB
PD	1606	F	45	12	CING	IHC
PD/AD/LB variant	1686	F	73	18	CING	IHC
DLB/AD	1699	F	89	7	CING	IHC
PD/AD/DLB	1864	M	63	24	CING	IHC
PD/DLB	1828	F	86	16	PUT	WB
PD	1938	M	70	26	PUT	WB
PD/DLB	1981	F	88	19.5	PUT	WB
PD/DLB	2003	F	95	12	PUT	WB
PD/dementia	2019	M	83	16.5	PUT	WB
PD/dementia	2053	M	75	6	PUT	WB
PD/dementia	2071	F	85	9	PUT	WB

AD, Alzheimer's disease; CING, cingulate cortex; DLB, dementia with Lewy bodies; LB, Lewy body; IHC, immunohistochemistry; PMD, postmortem delay; PUT, caudate putamen; WB, western blot.

**Table 2.** Clinical details of human frontal cortex tissue used for biochemical analysis (from QSBB)

Subject	Gender	Age (years)	PMD (h)	Pathology
Control 1	F	85	37	N/A
Control 2	M	93	112	N/A
Control 3	F	91	98.5	N/A
Control 4	M	87	36	N/A
iPD 1	F	69	52.5	Limbic
iPD 2	M	70	61.2	Limbic
iPD 3	F	87	47.45	Limbic
iPD 4	M	75	48	Limbic
G2019S 1	F	80	44.4	Limbic
G2019S 2	F	81	15	Limbic
G2019S 3	F	84	32.2	Limbic
G2019S 4	F	72	24.55	Limbic

iPD, idiopathic Parkinson's disease; Limbic, limbic subtype of Lewy body pathology according to McKeith consensus criteria for the classification of DLB; N/A, non-applicable; PMD, postmortem delay.

### D620n VPS35 exhibits normal stability, vesicular localization and sorting of retromer cargo

**Figure 2.**
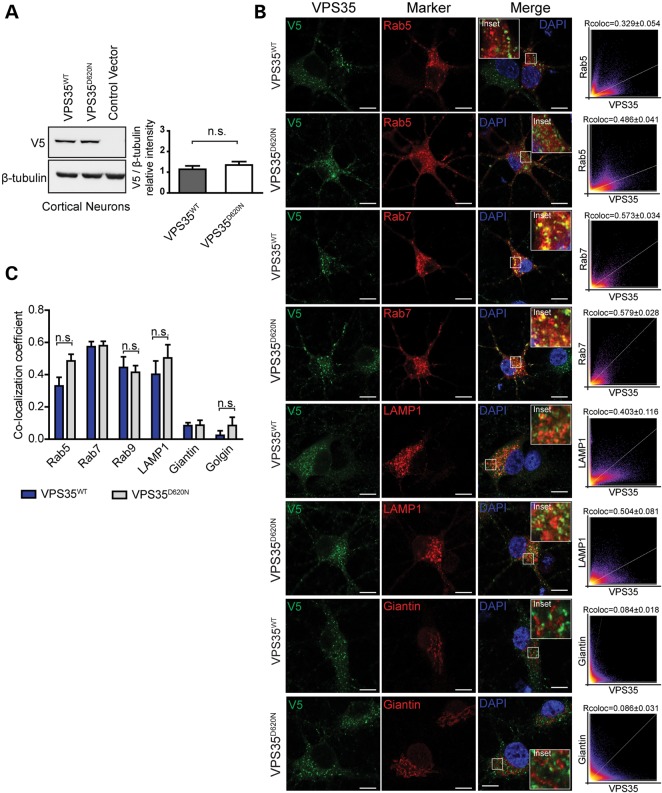
Normal steady-state levels and vesicular localization of VPS35^D620N^ in cortical neurons. (**A**) Western blot analysis of soluble extracts from primary cortical neurons infected with lentiviral vectors expressing V5-tagged human VPS35 (WT or D620N) or control virus with anti-V5 or β-tubulin antibodies. Densitometric analysis of human VPS35 normalized to β-tubulin levels indicates the equivalent expression of WT and D620N variants (mean ± SEM, *n* = 4 experiments). n.s., non-significant by unpaired, two-tailed Student's *t*-test. (**B**) Representative confocal microscopic images of primary cortical neurons co-labeled for WT or D620N human VPS35 (V5) and RFP-Rab5, GFP-Rab7, RFP-LAMP1 or *trans*-Golgi protein Giantin, and DAPI. Inset indicates enlarged boxed area in merged images. Cytofluorograms and correlation coefficients (Rcoloc, mean ± SEM, *n* ≥ 5 neurons) indicate the degree of co-localization of fluorescence signals for V5 and each marker. Scale bars: 10 μm. (**C**) Graph showing co-localization coefficients (mean ± SEM, *n* ≥ 5 neurons/group) of WT or D620N VPS35 with each vesicular marker in cortical neurons. n.s., non-significant by unpaired, two-tailed Student's *t*-test as indicated.

To explore the putative pathogenic effects of dominant familial PD mutations in *VPS35*, we generated lentiviral vectors expressing V5-tagged human VPS35 harboring the common D620N mutation or wild-type (WT) protein. The D620N mutation does not influence the steady-state levels of human VPS35 protein exogenously expressed in rat primary cortical neurons (Fig. 2A). Furthermore, the D620N mutation fails to significantly alter the vesicular localization of human VPS35 (Fig. 2B–C). WT and D620N variants of VPS35 display a similar degree of co-localization with multiple vesicular or membranous compartments, including early (Rab5), late (Rab7) and recycling (Rab9) endosomes, lysosomes (LAMP1) and the *trans*-Golgi network (Giantin/GOLGB1 and Golgin/GOLGA4) in primary cortical neurons (Fig. 2B–C). We do not observe any clear differences in the subcellular distribution of VPS35-positive endosomal and lysosomal vesicles between the WT and D620N variants (Fig. 2B). In general, the D620N mutation does not compromise the protein stability or vesicular localization of VPS35.

**Figure 3.**
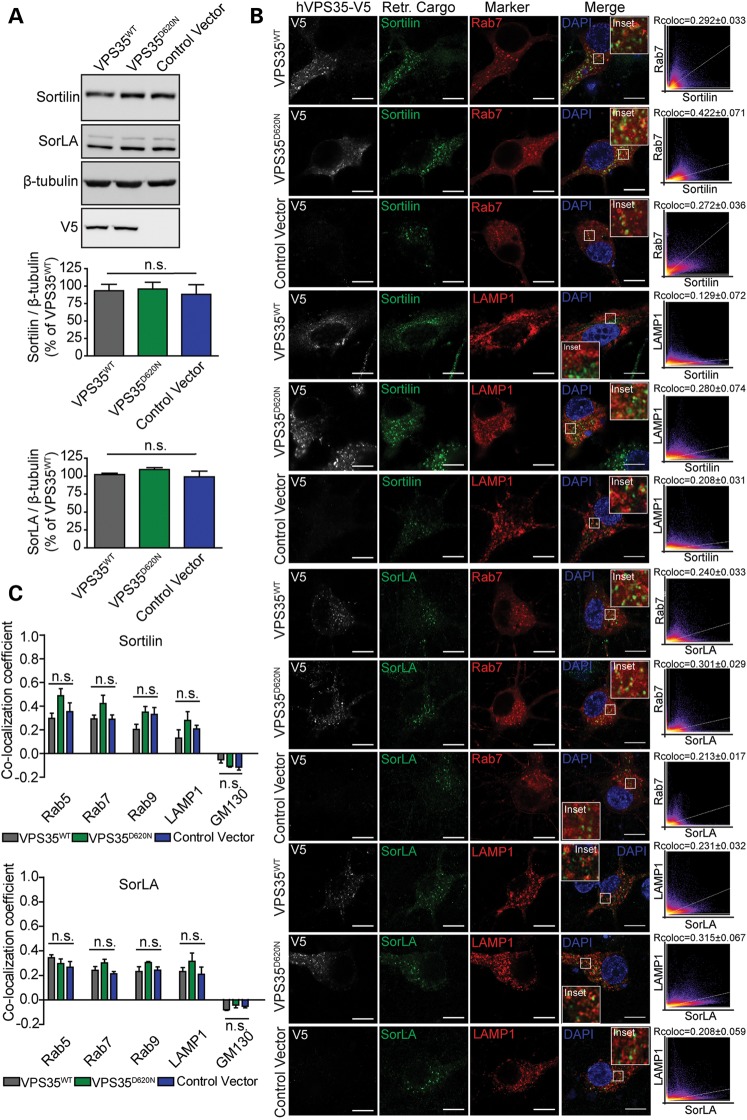
Protein levels and vesicular sorting of the retromer cargo sortilin and SorLA are not altered by VPS35^D620N^ expression in primary cortical neurons. (**A**) Western blot analysis of soluble extracts from primary cortical neurons infected with lentiviral vectors expressing V5-tagged human VPS35 (WT or D620N) or a control virus, with antibodies to sortilin, SorLA and V5 or β-tubulin as a protein loading control. Graphs indicate densitometric analysis of sortilin or SorLA normalized to β-tubulin levels and expressed as percent of the VPS35^WT^ condition (mean ± SEM, *n* = 4 experiments). (**B**) Representative confocal microscopic images of primary cortical neurons co-labeled for human VPS35 (V5), sortilin or SorLA and each vesicular marker (GFP-Rab7 or RFP-LAMP1), and DAPI. Inset indicates enlarged boxed area in merged images. Cytofluorograms and correlation coefficients (Rcoloc, mean ± SEM, *n* ≥ 7 neurons) indicate the degree of co-localization of fluorescence signals for sortilin or SorLA and each vesicular marker. Scale bars: 10 μm. (**C**) Graph showing co-localization coefficients (mean ± SEM, *n* ≥ 7 neurons/group) of sortilin (upper) or SorLA (lower) with each vesicular marker (RFP-Rab5, GFP-Rab7, GFP-Rab9, RFP-LAMP1 or Golgi protein GM130) in cortical neurons expressing human VPS35 (WT or D620N) or a control vector. n.s., non-significant by one-way ANOVA with Newman–Keuls *post hoc* analysis.

**Figure 4.**
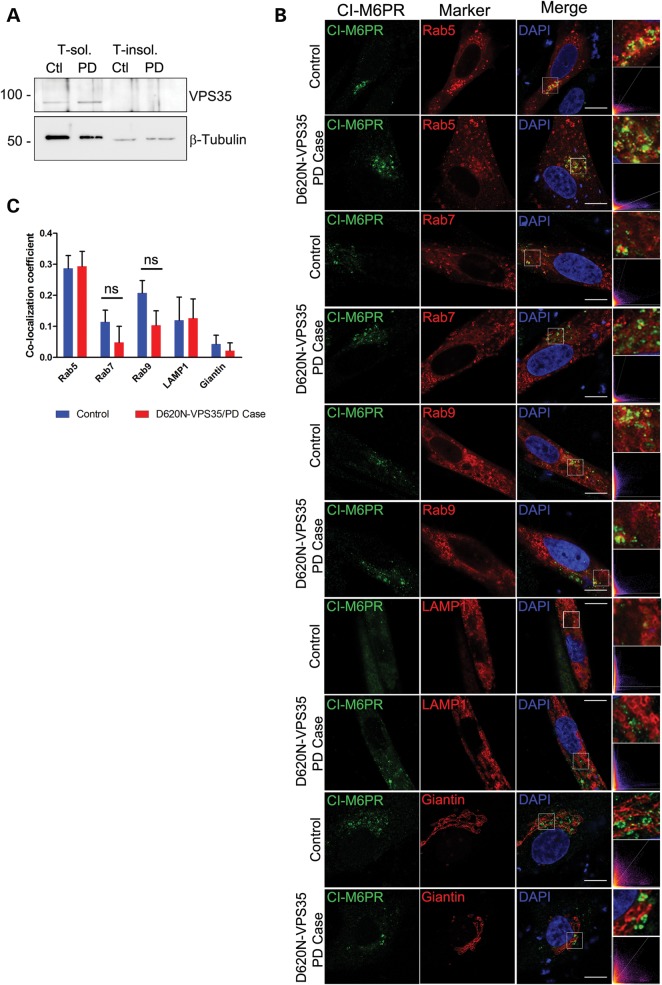
Normal VPS35 Levels and Vesicular Sorting of the Retromer Cargo CI-M6PR in Primary Human Fibroblasts Derived from a D620N Mutant PD Subject. (**A**) Western blot analysis of 1% Triton-soluble (T-sol.) and Triton-insoluble (T-insol.) fractions of primary fibroblasts derived from a Parkinson's disease subject harboring the D620N VPS35 mutation (PD) and a healthy control (Ctl). Blots are probed with antibodies to VPS35 and β-tubulin as a protein loading control. Molecular mass is indicated in kDa. (**B**) Representative confocal microscopic images and cytofluorograms of human primary fibroblasts (control or D620N) co-labeled for cation-independent mannose-6-phosphate receptor (CI-M6PR) and vesicular markers (RFP-Rab5, GFP-Rab7, GFP-Rab9, RFP-LAMP1 or trans-Golgi protein Giantin). Enlarged boxed areas of merged images are shown. Scale bar: 10 μm. (**C**) Graph indicates the co-localization coefficients (mean ± SEM, *n* ≥ 5 cells) of CI-M6PR with each vesicular marker in primary fibroblasts from control or D620N PD subjects. n.s., non-significant by unpaired, two-tailed Student's *t*-test, as indicated.

To determine whether the D620N mutation interferes with the vesicular sorting of retromer cargo proteins, we assessed the vesicular localization of sortilin, SorLA and CI-M6PR. The steady-state levels of sortilin and SorLA in cortical neurons are not altered by the overexpression of human WT or D620N VPS35 (Fig. 3A). Furthermore, the localization of endogenous sortilin and SorLA to endosomes (Rab5, Rab7 and Rab9), lysosomes (LAMP1) and the Golgi network (GM130) in cortical neurons are not significantly altered by the overexpression of human WT or D620N VPS35 (Fig. 3B–C). The expression of a third retromer cargo, CI-M6PR, could not be detected in cortical neurons. Patient-derived skin fibroblasts were next used to determine the effects of the D620N mutation on endogenous VPS35. Primary fibroblasts derived from a single PD subject harboring the D620N mutation reveal normal steady-state levels of endogenous VPS35 compared with WT control fibroblasts by western blot analysis (Fig. 4A). The localization of the retromer cargo, CI-M6PR, to endosomes (Rab5, Rab7 or Rab9), lysosomes (LAMP1) or the *trans*-Golgi network (Giantin) is not significantly altered in D620N mutant fibroblasts compared with WT control cells (Fig. 4B–C). The expression of additional retromer cargo, sortilin and SorLA, could not be detected in primary fibroblasts. Our data indicate that the D620N mutation in VPS35 does not adversely influence the vesicular sorting of retromer cargo proteins (i.e. sortilin, SorLA and CI-M6PR) in primary neurons or patient-derived fibroblasts.

### Familial PD mutations in VPS35 do not cause a loss-of-function in yeast

**Figure 5.**
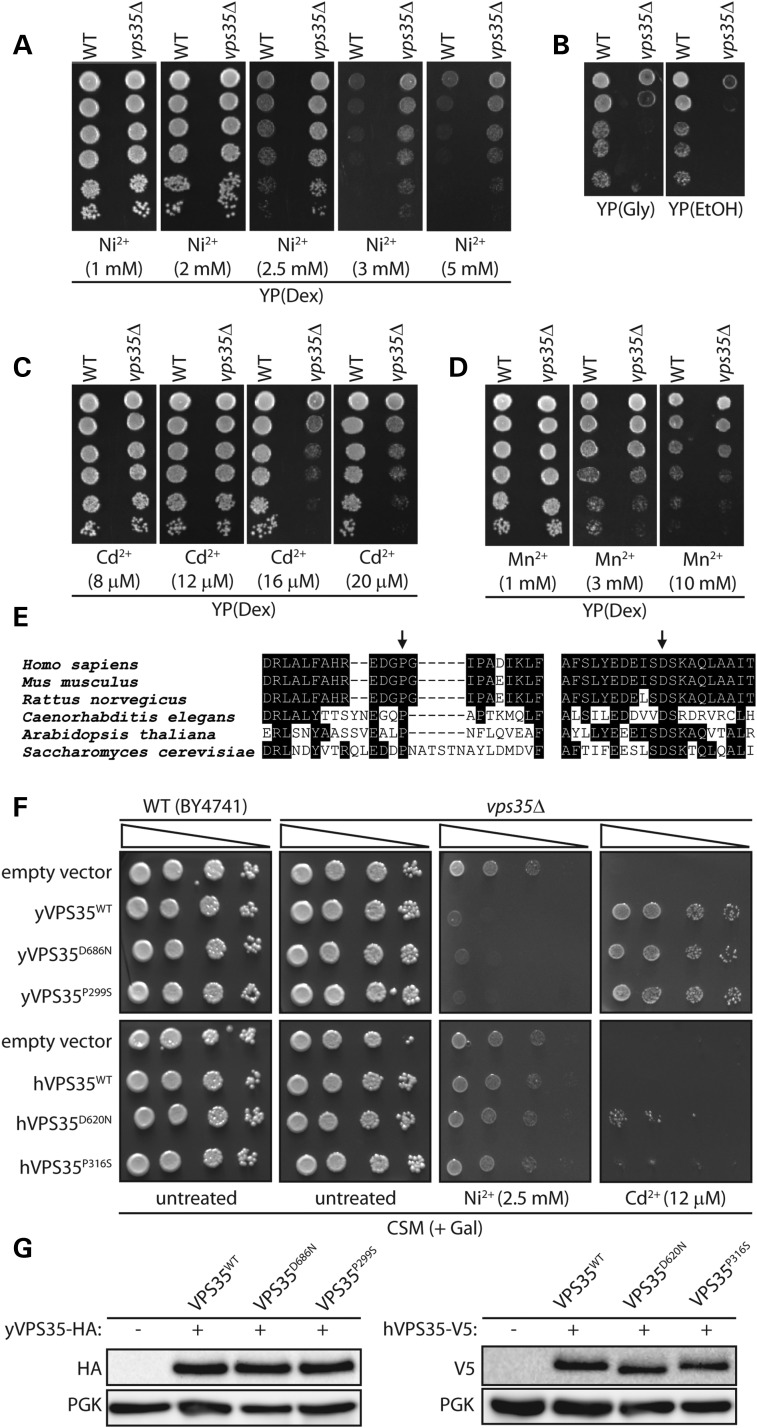
Analogous PD-like mutations in yeast VPS35 can functionally complement growth phenotypes in VPS35 null yeast cells. (**A–D**) Haploid yeast cells (BY4741, MATa), either wild-type (WT) or with a deletion of VPS35 (vps35Δ), were spotted onto YP(Dex) rich media containing different concentrations of (A) nickel (Ni^2+^), (C) cadmium (Cd^2+^) or (D) manganese (Mn^2+^), or YP media containing (B) non-fermentable carbon sources, glycerol (Gly) or ethanol (EtOH), and grown for 2–3 days at 30°C. Shown are 5-fold serial dilutions (from top to bottom) starting with equal numbers of cells. (**E**) Protein sequence alignment of VPS35 in the region encompassing the human P316 and D620 residues across several model species, indicating the high conservation of these two residues. (**F**) WT or vps35Δ yeast strains were transformed with galactose-inducible low-copy expression vectors containing HA-tagged yeast VPS35 (WT, D686N or P299S) or V5-tagged human VPS35 (WT, D620N or P316S) variants, or with an empty vector (p416GAL1). Equivalent numbers of cells were spotted as five-fold serial dilutions (from left to right) onto complete synthetic media lacking uracil (CSM) and containing galactose as the sole carbon source with or without additional Ni^2+^ (2.5 mM) or Cd^2+^ (12 µM), as indicated. (**G**) Western blot analysis of soluble extracts from WT yeast cells transformed with yeast or human VPS35 variants following growth on CSM-URA media containing galactose to induce VPS35 expression. VPS35 is detected using anti-HA or anti-V5 antibodies with an antibody to 3-phosphoglycerate kinase (PGK) used as a protein loading control.

To determine whether familial PD mutations in human VPS35 act through a gain-of-function or a loss-of-function mechanism, we employed the baker's yeast *S. cerevisiae* for functional complementation studies. Yeast contains a highly conserved ortholog of human VPS35 of 944 amino acids with ∼54% protein similarity (14). To initially identify phenotypes in yeast resulting from VPS35 loss-of-function, haploid yeast cells harboring a deletion of the endogenous *VPS35* gene (*Δvps35*) were evaluated for growth fitness on a number of carbon sources as well as sensitivity to heavy metal exposure, based on phenotypic data deposited in the *Saccharomyces* Genome Database (www.yeastgenome.org). *VPS35* null yeast exhibit increased resistance to growth on media containing nickel (Ni^2+^), increased sensitivity to cadmium (Cd^2+^) but no difference to manganese (Mn^2+^) compared with a WT yeast strain (Fig. 5A–D). *VPS35* null yeast further display normal growth on media containing fermentable carbon sources (dextrose or galactose) but reduced growth on non-fermentable carbon sources (glycerol and ethanol) compared with WT yeast (Fig. 5A–D). Non-fermentable carbon sources require mitochondrial oxidative phosphorylation for their utilization suggesting that deletion of *VPS35* in yeast may impair mitochondrial respiration.

The overexpression of V5-tagged human VPS35 variants [WT, D620N and P316S, a putative pathogenic mutation identified in a US family (6)] from a low-copy galactose-inducible vector (p416GAL1) is unable to robustly complement the growth phenotypes of *Δvps35* yeast on media containing Ni^2+^ or Cd^2+^ (Fig. 5F). However, we observe a rather modest rescue of the Cd^2+^-induced growth deficit in *Δvps35* yeast by expression of D620N VPS35, suggesting a partial complementation compared with WT or P316S VPS35 (Fig. 5F). Notably, the inducible overexpression of human VPS35 variants in WT or *Δvps35* yeast does not generally influence cell growth and therefore VPS35 variants are not intrinsically toxic to yeast (Fig. 5F). Our data suggest that human VPS35 lacks functional conservation with yeast VPS35, at least in regulating the susceptibility to heavy metal exposure.

As an alternative strategy, mutations analogous to the familial PD variants, D686N (D620N in hVPS35) and P299S (P316S in hVPS35), were introduced into yeast VPS35 in low-copy galactose-inducible expression vectors to assess the functional effects of these analogous PD-like mutations. The D620 and P316 residues in VPS35 are highly conserved from yeast to humans (Fig. 5E). The overexpression of HA-tagged yeast VPS35 variants (WT, D686N and P299S) are able to equivalently and robustly rescue the growth phenotype of *Δvps35* yeast on media containing Ni^2+^ or Cd^2+^, whereas the overexpression of yeast VPS35 variants does not influence the normal growth of WT or *Δvps35* yeast strains (Fig. 5F). Western blot analysis reveals equivalent levels of expression of human and yeast VPS35 variants in yeast following galactose induction (Fig. 5G). Collectively, our data indicate that analogous PD-like mutations in yeast VPS35 (P299S and D686N), that are highly conserved with human VPS35 (P316S and D620N) (Fig. 5E), are fully functional compared with the WT protein, suggesting that these dominantly inherited mutations do not act through a loss-of-function mechanism. Our data support instead a gain-of-function mechanism for the pathogenic actions of familial PD mutations in VPS35.

### Human VPS35 induces neuronal cell death and increases neuronal vulnerability to stress

**Figure 6.**
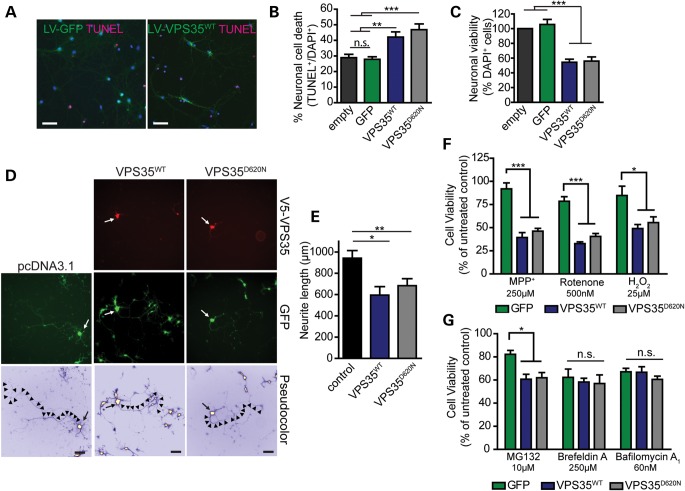
Overexpression of human VPS35 induces neuronal cell death, impairs neurite outgrowth and increases neuronal vulnerability to cellular stress. (**A**) Representative immunofluorescent images of primary cortical neurons at DIV 14 infected with GFP or VPS35^WT^ lentivirus (green) and co-labeling of apoptotic (TUNEL-positive, red) or total (DAPI-positive, cyan) nuclei to assess neuronal cell death. Scale bar: 50 μm. (**B**) Cortical neurons infected with lentiviral vectors expressing GFP or human VPS35 variants were assessed for apoptotic cell death by quantifying the number of TUNEL-positive neurons as a percent of total neurons (DAPI-positive nuclei) for each condition (mean ± SEM, *n* = 5 independent cultures). (**C**) Neuronal viability was assessed in cortical cultures infected with lentiviral vectors at DIV 14 by counting the average number of neurons (DAPI-positive nuclei) for each condition, and expressed as a percent of an empty control virus (mean ± SEM, *n* = 5 independent cultures). (**D**) Representative fluorescent microscopic images of cortical neurons at DIV 7 co-labeled with V5-tagged human VPS35 (WT or D620N) and GFP. GFP images were pseudocolored with ICA to identify neuronal soma (arrowhead) and axonal processes (arrows) for neurite length measurements. Scale bar: 200 μm. (**E**) Quantitation of axonal process length from GFP-positive neurons expressing human VPS35 (WT or D620N) compared with control neurons (empty vector). Bars represent neurite length (mean ± SEM, *n* = 5 independent cultures) from 170–189 neurons. (**F**) Primary cortical neurons infected with lentiviral vectors expressing human VPS35 (WT or D620N) or GFP were treated at DIV 14 with cellular toxins (MPP^+^, rotenone or H_2_O_2_) for 24 h and assessed by alamarBlue cell viability assay. Bars represent cell viability expressed as a percent of untreated cultures for each condition (mean ± SEM, *n* ≥ 3 independent cultures). (**G**) Cortical neurons infected with lentiviral vectors as in F were treated with cellular toxins (MG132, Brefeldin A or Bafilomycin A1) for 24 h prior to cell viability assays. Cell viability relative to untreated cultures is shown (mean ± SEM, *n* ≥ 3 independent cultures). Data were analyzed by one-way ANOVA with Newman–Keuls *post hoc* analysis, as indicated (*P<0.05, **P<0.01 or ***P<0.001). n.s., non-significant.

To explore the putative pathogenic effects of familial PD mutations in *VPS35*, human VPS35 variants (WT and D620N) were overexpressed in neuronal cultures to model gain-of-function effects. We evaluated the viability of rat primary cortical neurons following infection with lentiviral vectors expressing human VPS35 variants or GFP as a negative control. Lentiviral infection of cortical cultures at days-*in vitro* (DIV) 3 results in the efficient and long-term transduction of the majority of cortical neurons as indicated by labeling of cultures for V5-tagged VPS35 or GFP (Fig. 6A). The overexpression of WT or D620N VPS35 in cortical neurons up to DIV 14 significantly increases apoptotic neuronal cell death (Fig. 6B) and reduces neuronal viability (Fig. 6C) compared to infection with control lentiviral vectors (empty or GFP). Furthermore, the transient overexpression of human WT or D620N VPS35 in cortical neurons significantly impairs neurite outgrowth (Fig. 6D–E). Notably, no significant differences are observed between WT and D620N variants upon these VPS35-dependent neuronal phenotypes.

To further explore and compare the impact of human WT and D620N VPS35 on neuronal vulnerability, we assessed the contribution of VPS35 to neuronal cell death induced by various cellular toxins or stressors implicated in the pathogenesis of PD (5). The lentiviral-mediated overexpression of human WT or D620N VPS35 significantly sensitizes to neuronal cell death induced by exposure of primary cortical cultures to the mitochondrial Complex-I inhibitors, MPP^+^ (250 µM) and rotenone (500 nM), and to oxidative stress induced by hydrogen peroxide (25 µM) (Fig. 6F). Human VPS35 variants also increase the vulnerability of cortical neurons to proteosomal inhibition induced by MG132 (10 µM) although to a smaller extent (Fig. 6G). VPS35 overexpression fails to increase neuronal vulnerability to the autophagy/lysosomal inhibitor, bafilomycin A1 (60 nM), or to disruption of ER-Golgi transport induced by brefeldin A (250 µM) (Fig. 6G), thereby supporting a specific interaction of VPS35 with neuronal susceptibility to mitochondrial toxins, oxidative stress and proteasomal inhibition. No significant differences are observed between the WT and D620N variants of VPS35 in their capacity to increase neuronal vulnerability to toxins thereby arguing against a loss-of-function mechanism for familial PD mutations. Taken together, our data demonstrate that the overexpression of human VPS35 induces neuronal cell death, impaired neurite outgrowth and increases neuronal vulnerability to cellular stress most likely through a gain-of-function mechanism. However, pathogenic phenotypes induced in primary neuronal culture models do not obviously permit the discrimination between the effects of WT and D620N VPS35, an observation that is similar for dominantly inherited mutations in α-synuclein (15).

### Viral-mediated expression of D620N VPS35 induces dopaminergic neurodegeneration in rats

**Figure 7.**
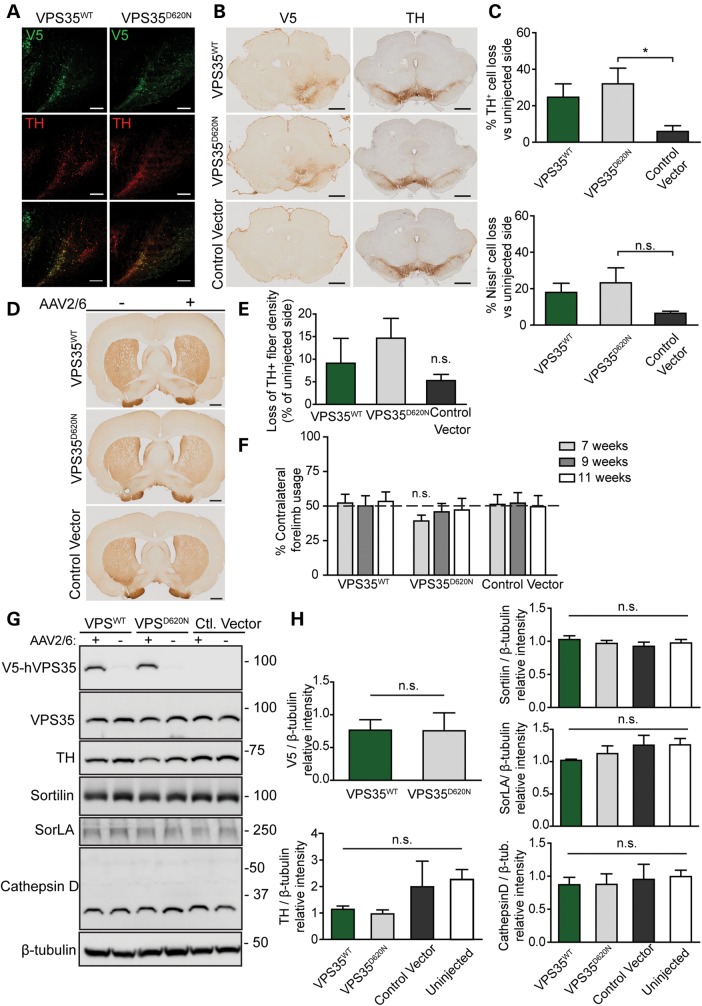
Dopaminergic neuronal degeneration induced by AAV2/6-mediated expression of D620N VPS35 in the substantia nigra of adult rats. (**A**) Photomicrographs showing immunofluorescent co-labeling of substantia nigra with anti-V5 and anti-TH antibodies indicating the equivalent expression of V5-tagged human VPS35^WT^ and VPS35^D620N^ proteins within dopaminergic neurons at 12 weeks following stereotactic injection of AAV2/6 vectors. Scale bar: 200 μm. (**B**) Representative photomicrographs of anti-V5 and anti-TH immunohistochemical analysis in adjacent sections of rat substantia nigra at 12 weeks following the stereotactic injection of AAV2/6 vectors expressing human VPS35^WT^ and VPS35^D620N^ or a control vector. V5 labeling in the substantia nigra is accompanied by a decrease in TH immunostaining. Scale bar: 1 mm. (**C**) Stereological analysis of TH-positive dopaminergic or total Nissl-positive neurons in the substantia nigra expressed as percent cell loss relative to the contralateral uninjected nigra. Bars represent mean ± SEM (*n* = 6 animals/group). *P<0.05 by one-way ANOVA with Dunnett's *post hoc* analysis. n.s., non-significant. (**D**) Representative photomicrographs of immunostaining for TH-positive nerve terminal in the striatum at 12 weeks following AAV2/6 delivery. Scale bar: 1 mm. (**E**) Quantitation of optical density of TH immunostaining in the striatum. Data are expressed as percent loss of TH-positive fibers relative to the contralateral uninjected side. Bars represent the mean ± SEM (*n* ≥ 4 animals/group). Data were assessed by one-way ANOVA with Dunnett's *post hoc* analysis. (**F**) Cylinder asymmetry test performed at 7, 9 and 11 weeks postsurgery assessing forelimb contacts with the cylinder wall over a 5 min period. Forelimb use contralateral to the injected nigra is expressed as a percent of total forelimb contacts (left + right) on the cylinder wall. Dashed line indicates the expected forelimb usage under normal conditions. Bars represent mean ± SEM (*n* = 8 animals/group). Data were assessed by one-way ANOVA with Newman–Keuls *post hoc* analysis. (**G**) Western blot analysis of soluble extracts from ventral midbrain at 12 weeks following stereotactic injection of rats with AAV2/6 vectors, separated into ipsilateral (+) and contralateral (−) hemispheres. Blots were probed with antibodies to V5, VPS35, TH, sortilin, SorLA and cathepsin D, with β-tubulin as a loading control. Molecular masses are indicated in kDa. (**H**) Densitometric analysis of V5, TH, sortilin, SorLA or cathepsin D levels normalized to β-tubulin levels on blots containing ventral midbrain extracts. Bars represent mean ± SEM (*n* = 3–4 animals/group). Data were analyzed by one-way ANOVA with Newman–Keuls *post hoc* analysis for multiple comparisons, or by unpaired, two-tailed Student's *t*-test for pair-wise comparisons, as indicated. n.s., non-significant.

To explore the pathogenic effects of dominant *VPS35* mutations *in vivo*, human WT and D620N VPS35 were overexpressed in substantia nigra dopaminergic neurons of rats to develop an animal model of *VPS35*-associated PD. Adeno-associated viral vectors (AAV2/6) expressing V5-tagged human VPS35 (WT or D620N) were delivered to the substantia nigra pars compacta of adult rats by unilateral stereotactic injection. At 12 weeks postinjection of virus, we observe robust, widespread and equivalent expression of WT and D620N VPS35 throughout the injected substantia nigra with substantial co-localization with tyrosine hydroxylase (TH)-positive dopaminergic neurons (Fig. 7A–B). To assess whether human WT or D620N VPS35 induces dopaminergic neurodegeneration, we quantified the number of TH-positive dopaminergic and total Nissl-positive neurons in the substantia nigra pars compacta of injected animals using unbiased stereological methodology. The expression of D620N VPS35 produces a significant loss of nigral dopaminergic neurons (32.02 ± 8.6% loss) in the injected nigra compared with a control virus (5.9 ± 3.2% loss) (Fig. 7C). The expression of WT VPS35 induces an intermediate level of dopaminergic neuronal loss (24.6 ± 7.4% loss) in the injected nigra compared with the control condition (Fig. 7C). A parallel loss of Nissl-positive neurons is observed in the injected substantia nigra relative to the non-injected nigra with each virus (D620N, 23.23 ± 8.2%; WT, 17.93 ± 5.1%; control, 6.5 ± 1.1%) indicating dopaminergic neurodegeneration rather than a loss of TH expression or dopaminergic phenotype (Fig. 7C). A corresponding loss of TH-positive dopaminergic nerve terminals (D620N, 14.6 ± 4.4%; WT, 9.1 ± 5.5%; control, 5.3 ± 1.3%) is also observed in the ipsilateral striatum compared with the contralateral striatum of rats injected with each virus (Fig. 7D–E). To determine whether VPS35-induced dopaminergic neuronal loss culminates in motoric deficits in rats, we employed the cylinder test to measure contralateral forelimb use which is controlled by the ipsilateral substantia nigra (16). However, we observe no significant impairment of contralateral forelimb use induced by the ipsilateral expression of human WT or D620N VPS35 at 7, 9 and 11 weeks postinjection of virus (Fig. 7F).

To confirm the equivalent expression of human VPS35 variants in injected animals, we conducted western blot analysis of ventral midbrain extracts at 12 weeks postinjection. Human V5-tagged WT and D620N VPS35 are detected at equivalent levels in the injected ventral midbrain of rats (Fig. 7G–H). An antibody that detects both rat and human VPS35 fails to demonstrate the overexpression of human VPS35 relative to endogenous VPS35 in ventral midbrain extracts suggesting either a dilution effect of the transgene in this brain region or a potential lowering of endogenous VPS35 levels in response to human VPS35 expression (Fig. 7G). Western blot analysis further indicates a reduction of TH levels in the ventral midbrain induced by WT and D620N VPS35 expression (Fig. 7G–H), which correlates with the observed dopaminergic neuronal loss (Fig. 7C). Consistent with our data in neuronal cultures (Fig. 3A), we do not observe alterations in the steady-state levels of sortilin or SorLA resulting from the expression of human VPS35 variants in the ventral midbrain of rats (Fig. 7G–H). It was not possible to reliably detect CI-M6PR in these ventral midbrain extracts with currently available antibodies. Instead, the lysosomal protease cathepsin D, a soluble ligand of CI-M6PR (17), was assessed. However, the expression of human VPS35 variants does not alter the steady-state levels or processing of cathepsin D in the ventral midbrain (Fig. 7G–H).

**Figure 8.**
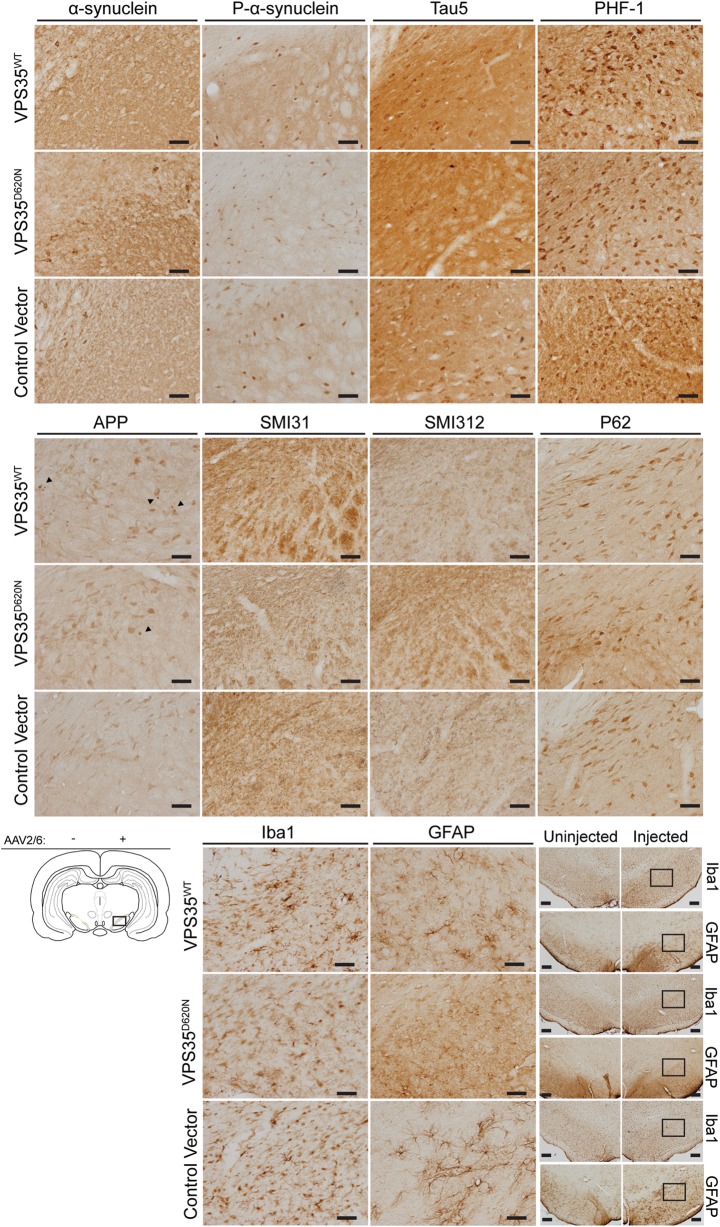
AAV2/6-mediated expression of human VPS35 in the rat substantia nigra induces axonal pathology*.* Immunohistochemical analysis of substantia nigra at 12 weeks following the stereotactic injection of AAV2/6 vectors expressing human VPS35^WT^ and VPS35^D620N^ or a control vector. Antibodies detecting total α-synuclein, α-synuclein phosphorylated at Ser129 (P-α-synuclein), total tau (Tau5), tau phosphorylated at Ser396 and Ser404 (PHF-1), APP isoforms, phosphorylated neurofilament H (SMI31), phosphorylated neurofilament M/H (SMI312), autophagy substrate p62/sequestosome 1, GFAP and Iba1 were employed. Representative photomicrographs are taken from the injected substantia nigra as indicated in the schematic diagram. Scale bar: 50 μm. Macroscopic images of GFAP and Iba1 in the injected and non-injected substantia nigra are shown for comparison. Boxes indicate the region used for high-power images of the injected substantia nigra. Scale bar: 50 μm.

The neuropathological spectrum of PD brains harboring *VPS35* mutations is uncertain since no mutation carriers have yet come to autopsy (9). To determine the pathological consequences of *VPS35* mutations *in vivo*, we assessed the distribution of a number of classical markers of neurodegeneration in the substantia nigra pars compacta of rats expressing WT or D620N VPS35. At 12 weeks postinjection, we do not observe alterations in the distribution of total or phosphorylated (Ser129) α-synuclein (Fig. 8), a major component of Lewy body pathology in PD and other α-synucleinopathies (3,18), following human VPS35 expression. Similarly, we do not detect changes in total (Tau5) or hyperphosphorylated (PHF-1) forms of the microtubule-associated protein tau (Fig. 8), a protein involved in neurodegenerative tauopathies (19) and implicated in PD (20,21). Alterations in phosphorylated neurofilaments (SMI31 and SMI312), a marker of axonal processes and terminals, or p62/sequestosome 1, a marker of autophagic/lysosomal vacuoles, are also not detected in the injected substantia nigra (Fig. 8). The expression of human WT and D620N VPS35 results in the accumulation of spherical inclusions containing amyloid precursor protein (APP) within axonal processes (Fig. 8), a sensitive marker of axonal damage (22). Gliosis typically accompanies neuronal degeneration in PD and other neurodegenerative diseases. However, astrogliosis (GFAP) and microgliosis (Iba1) are not generally observed in the substantia nigra of rats at 12 weeks postinjection of virus (Fig. 8).

**Figure 9.**
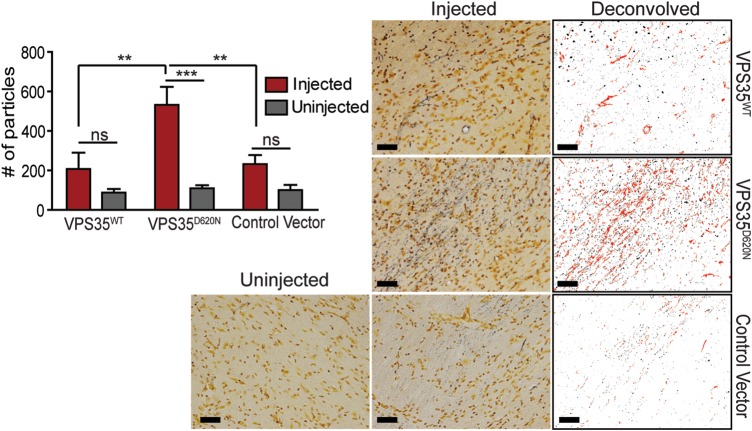
Nigral axonal degeneration induced by AAV2/6-mediated expression of D620N VPS35. Representative photomicrographs of substantia nigra sections processed with Gallyas silver stain, from rats injected with AAV2/6 vectors expressing human VPS35^WT^ and VPS35^D620N^ or a control vector, to reveal degenerating axons and nerve terminals (indicated in black). Scale bar: 50 μm. Images were subjected to deconvolution, color separation and filtering (circularity index ≤0.6 to remove nuclei/soma) using ImageJ to isolate black particles representative of neuritic processes and terminals for quantitation. Examples of particles analyzed are shown pseudocolored in red. Data are expressed as the number of black particles (silver-positive neurites/terminals) and bars represent the mean ± SEM (*n* = 6 animals/group). **P<0.01 or ***P<0.001 by one-way ANOVA with Tukey's *post hoc* analysis as indicated. n.s., non-significant.

The accumulation of APP-positive axonal inclusions suggests that human VPS35 expression may also promote axonal degeneration. Substantia nigra sections from injected rats at 12 weeks were stained with silver which specifically labels components of degenerating neurons, including lysosomes, axonal processes and their terminals (23,24). The expression of D620N VPS35 induces a significant increase in the appearance of silver-positive degenerating axonal processes and nerve terminals compared with WT VPS35 or a control virus (Fig. 9). Collectively, our data reveal the marked degeneration of substantia nigra dopaminergic neurons and axonal pathology induced by the expression of the familial D620N mutation of VPS35 in the adult rat brain, which recapitulates a cardinal pathological hallmark of PD.

## DISCUSSION

Mutations in the *VPS35* gene cause late-onset, autosomal dominant PD with the D620N mutation representing a common cause of *VPS35*-linked disease (6–9). The mechanism(s) through which dominant *VPS35* mutations precipitate neurodegeneration in PD are not known, and the neuropathological consequences of *VPS35* mutations in PD subjects are poorly defined at present. Here, we present multiple lines of evidence demonstrating that the dominant D620N mutation in *VPS35* is capable of inducing dopaminergic neuronal degeneration most likely through a gain-of-function mechanism. First, familial mutations do not compromise VPS35 protein stability or its normal localization to endosomes and lysosomes. Furthermore, the vesicular sorting of retromer cargo, sortilin, SorLA and CI-M6PR, which depend on VPS35 as a key component of the retromer substrate recognition subcomplex (11), is not altered by mutations in VPS35. Second, the equivalent PD-like mutation in yeast VPS35 (D686N) is fully functional compared with the WT yeast protein in complementing growth phenotypes in *VPS35* null yeast. The D620 residue and the VPS35 protein sequence in general are highly conserved throughout evolution. Therefore, our data suggest that the D620N mutation does not result in a loss-of-function. Third, the lentiviral-mediated overexpression of human VPS35 variants impairs neuronal integrity and viability, and increases the vulnerability of neurons to PD-relevant cellular stressors, including mitochondrial Complex-I inhibition, oxidative stress and proteasomal inhibition (5). Fourth, the AAV-mediated expression of human D620N VPS35 in the substantia nigra of adult rats induces marked dopaminergic neuronal degeneration with an intermediate effect of human WT VPS35 compared with a control virus. Therefore, the D620N mutation in VPS35 recapitulates a cardinal pathological hallmark of PD in a novel animal model. Taken together, our data demonstrate that the PD-associated dominant D620N mutation in *VPS35* is able to induce neuronal degeneration most likely through a gain-of-function mechanism, thereby establishing an important contribution of VPS35 to the development of PD.

The distinct absence of truncation, rearrangement or deletion mutations in *VPS35* identified so far across multiple PD cohorts worldwide, together with the dominant inheritance pattern of the common D620N mutation, suggests that mutations are not likely to act through a loss-of-function mechanism. Thus, *VPS35* mutations most likely operate through a gain-of-function mechanism. Consistent with such a mechanism, the D620N mutation does not compromise the steady-state levels of VPS35 and its normal localization to early and late endosomes and lysosomes within cortical neurons, suggesting the absence of major structural perturbations to VPS35. A recent study suggests a redistribution of early and late endosomes containing D620N VPS35 to the perinuclear region in A431 cells (25). However, our data do not indicate a redistribution of D620N VPS35-positive vesicles in cortical neurons. We further demonstrate that the ectopic expression of D620N VPS35 in rodent cortical neurons or endogenous D620N VPS35 in human fibroblasts does not influence the levels or vesicular sorting of well-known retromer cargo proteins, such as sortilin, SorLA and CI-M6PR. Thus, the D620N mutation does not apparently influence the overall substrate recognition or binding of retromer cargo to VPS35. A recent study suggests that the expression of D620N VPS35 causes a defect in the vesicular trafficking and processing of the lysosomal protease cathepsin D, a soluble ligand of CI-M6PR (25). Whether the defective trafficking of cathepsin D induced by D620N VPS35 also occurs in neurons and promotes neuronal degeneration in PD remains unclear. However, we failed to observe alterations in the levels and processing of cathepsin D in the ventral midbrain of rats expressing human D620N VPS35. Our findings suggest that familial mutations in *VPS35* do not lead to an overall disruption of VPS35 protein structure and retromer-dependent protein sorting in neurons, implying that mutations may instead exert subtle or selective effects on specific retromer cargo and/or in specific cell types.

Recent studies have suggested that the D620N mutation might compromise neuroprotective effects mediated by human VPS35 overexpression, consistent with a loss-of-function mechanism. WT VPS35 was recently shown to protect against neuronal toxicity induced by the mitochondrial Complex-I inhibitor, MPP^+^ (26), as well as against G2019S LRRK2 overexpression or RAB7L1 deficiency (27), two models relevant to familial PD. In this scenario, *VPS35* haploinsufficiency would be sufficient to induce neurodegeneration related to PD. While the homozygous deletion of *VPS35* in mice results in early embryonic lethality, *VPS35* haploinsufficiency has been reported to enhance Alzheimer's disease (AD)-like neuropathology in a mouse model of AD (28). Whether PD-related neuropathology or motor deficits occur in mice heterozygous for VPS35 is not yet known (28). We employed yeast as a model to demonstrate that mutations equivalent to D620N in a highly conserved yeast ortholog of VPS35 (i.e. D686N) can normally complement growth phenotypes in Δ*vps35* yeast thereby indicating that the D → N substitution at this highly conserved residue does not compromise VPS35 function. In contrast to prior studies (26,27), we demonstrate that the overexpression of human WT VPS35 in primary cortical neurons is detrimental rather than neuroprotective and leads to neuronal cell death and impaired neurite outgrowth. Moreover, VPS35 overexpression dramatically increased neuronal vulnerability to PD-relevant cellular stressors, including the mitochondrial Complex-I inhibitors MPP^+^ and rotenone, rather than eliciting neuroprotection as previously suggested (26). Importantly, the D620N mutation did not impair VPS35-induced neuronal toxicity or increased vulnerability which would be inconsistent with a loss-of-function mechanism. In our neuronal culture model we were not able to discern obvious differences between WT and D620N VPS35 in their capacity to induce neuronal damage. In contrast, the viral-mediated overexpression of human D620N VPS35 in the rat substantia nigra induced dopaminergic neuronal degeneration and axonal damage, whereas WT VPS35 produced an intermediate level of neuropathology. Therefore, the pathological effects of the D620N mutation can be unmasked given the proper cellular context, and supports a gain-of-function mechanism for the actions of PD-associated *VPS35* mutations.

We have developed a novel animal model of *VPS35*-associated PD that recapitulates one of the cardinal pathological features of disease, i.e. nigral dopaminergic neuronal loss. The degeneration of dopaminergic neurons in adult rats induced by D620N VPS35 was pronounced at 12 weeks after viral delivery. Whether this model recapitulates the progressive nature of neuronal loss in PD is not yet clear. It will be important in future studies to determine whether varying the dosage or time course of transgene expression would be sufficient to further enhance nigral neuronal degeneration, and potentially exacerbate differences between WT and D620N VPS35. The expression of D620N VPS35 in the rat substantia nigra does not recapitulate other aspects of PD-related neuropathology such as the formation of α-synuclein-positive Lewy pathology, tau-positive neurofibrillary tangles or gliosis. However, we do not yet know the full repertoire of pathological inclusions or aggregates in brains from PD subjects with *VPS35* mutations. Our *in vivo* studies formally prove that the dominant D620N mutation in *VPS35* can induce dopaminergic neuronal loss, a key pathological feature of familial and idiopathic PD. This new rodent model of *VPS35*-associated PD will prove useful for uncovering the molecular mechanisms of VPS35-dependent neurodegeneration and for the identification of disease-modifying therapeutic agents.

## MATERIALS AND METHODS

### Animals

Rats were maintained in a pathogen-free barrier facility and exposed to a 12 h light/dark cycle with food and water provided *ad libitum*. Pregnant female Sprague-Dawley rats were obtained from Charles River Laboratories (L'Arbresle Cedex, France) and resulting P1 rats were used for preparation of primary neuronal cultures. All animal experiments were approved by the SCAV (Service de la consommation et des affaires veterinaires) in the Canton de Vaud, Switzerland (Animal authorization No. 2572), and conducted in strict accordance with the European Union directive (2010/63/EU) for the care and use of laboratory animals.

### Expression plasmids and antibodies

A pLenti6/V5/DEST mammalian expression plasmid containing full-length human VPS35 with a C-terminal V5 tag was obtained from Addgene [#21691, (29)]. D620N and P316S mutations were introduced by PCR-mediated site-directed mutagenesis and sequenced to confirm their integrity. V5-tagged VPS35 cDNAs (WT, D620N or P316S) were amplified by PCR from a pLenti6-VPS35 vector, and cloned into a directional pENTR/D-TOPO entry vector (Invitrogen), sequenced to confirm their integrity, and subjected to recombination with a Gateway-compatible lentiviral vector, pSIN-PGK-WHV (30) (kindly provided by Dr. Nicole Deglon, CHUV, Switzerland). As plasmid controls, pcDNA3.1 (Invitrogen), pEGFP-N1 (Clontech) and pDsRed-Max-N1 (Addgene #21718) were employed. Expression plasmids containing human RFP-Rab5A (#14437), human GFP-Rab7A (#12605), human GFP-Rab9A (#12663) and rat LAMP1-RFP (#1817) were obtained from Addgene.

The following primary antibodies were used: rabbit VPS35 (ABT48, Millipore), mouse anti-V5 and anti-V5-peroxidase (Invitrogen), mouse anti-β-tubulin (clone TUB 2.1, Sigma-Aldrich), rabbit anti-tyrosine hydroxylase (TH, NB300-109, Novus Biologicals), mouse anti-TH (clone TH-2, Sigma-Aldrich), mouse anti-HA-peroxidase (clone 12CA5, Roche), mouse anti-PGK (clone 22C5D8, Invitrogen), rabbit anti-Dynamin-1 (PA1-660, Thermo Scientific), mouse anti-TIM23 (clone 32, BD Biosciences), mouse anti-α-synuclein (clone 42, BD Biosciences), mouse anti-pSer129 α-synuclein (Wako), mouse anti-GM130 (clone 35, BD Biosciences), rabbit anti-Giantin (ab24586, Abcam), rabbit anti-tGolgin-1/GOLGA4 (kindly provided by Dr Mickey Marks, University of Pennsylvania), rabbit anti-sortilin (ab16640, Abcam), rabbit anti-SorLA (ab16642, Abcam), mouse anti-CI-M6PR (clone 2G11, Abcam), rabbit anti-cathepsin D (Santa Cruz Biotech), rabbit anti-Iba1 (Wako), rabbit anti-GFAP (Z0334, Dako), mouse anti-phosphorylated neurofilament H (clone SMI-31, Covance), mouse anti-phosphorylated neurofilament M/H (clone SMI-312, Covance), mouse anti-synaptophysin 1 (clone 7.2, Synaptic Systems), guinea pig anti-p62 (GP62, Progen Biotech), mouse anti-APP (clone 22C11, Millipore), mouse anti-Tau (clone TAU-5, Invitrogen), mouse anti-PHF-1 (kindly provided by Dr Peter Davies, Albert Einstein College of Medicine). For bright-field microscopy, biotinylated secondary antibodies used were goat anti-mouse, anti-rabbit or anti-guinea pig (Vector Laboratories) followed by ABC reagent and DAB Peroxidase Substrate (Vector Laboratories). For fluorescence confocal imaging, secondary antibodies used were: AlexaFluor-488, -546, or -633 goat anti-mouse IgG (H + L), and AlexaFluor-488, -546 or -633 goat anti-rabbit IgG (H + L) (Life Technologies). Secondary antibodies used were: HRP- or biotin-conjugated mouse monoclonal anti-rabbit IgG, light chain-specific (Jackson Immunoresearch), goat anti-mouse IgG, light chain-specific (Jackson Immunoresearch) and goat polyclonal anti-guinea pig IgG, H + L (Abcam).

### Yeast growth assays

Human VPS35 variants (WT, D620N or P316S) were amplified by PCR from a pLenti6-VPS35 vector (Addgene #21691), and yeast VPS35 variants (WT, D686N or P299S) were amplified by PCR from a pBG1805-VPS35 vector containing a C-terminal HA tag (Thermo Scientific). Blunt-end PCR products were cloned into a directional pENTR/D-TOPO entry vector, sequenced to confirm their integrity, and subjected to recombination with the Gateway-compatible vector, p416GAL1-ccdB (CEN6, *URA3*) obtained from Addgene (31). Yeast haploid WT parental or *vps35Δ* strains on a BY4741 genetic background (*MATa*, *his3Δ1*, *leu2Δ0*, *met15Δ0*, *ura3Δ0*) were obtained from Thermo Scientific. Yeast cells were transformed with plasmids using a standard lithium acetate procedure. Yeast cells transformed with p416GAL1 expression plasmids were routinely grown in complete synthetic media lacking uracil (CSM-URA) with 2% dextrose. Equivalent yeast cells were serially diluted (5-fold) and spotted onto plates containing solid media (YP or CSM-URA) with 2% dextrose, galactose, glycerol or ethanol as the sole carbon source. Cells were grown at 30°C for 2–3 days before imaging.

### Lentivirus production

Lentiviral vectors were produced as described (32) in HEK-293T cells using a third generation packaging system. Lentivirus was purified by centrifugation and resuspended in 1× phosphate buffered saline pH 7.4 and 0.5% bovine serum albumin buffer. Viral titer was determined using the HIV-1 p24 antigen ELISA kit (Zeptometrix Corp, Buffalo, USA). A p24 of 66 ng/ml was routinely used for infecting primary neuronal cultures at a density of 400 000 cells in 3 ml media.

### Cell culture and transfection

HEK-293T cells were grown in Dulbecco's modified Eagle's media (DMEM) supplemented with 10% fetal bovine serum (FBS) and addition of penicillin/streptomycin in a humidified 37°C incubator with 5% CO_2_. Primary human fibroblasts were maintained in DMEM supplemented with 10% FBS, 2 mM L-glutamine and penicillin/streptomycin. Expression plasmids were transfected into cells using XtremeGENE HP DNA transfection reagent (Roche Applied Science) or Lipofectamine 2000 (Life Technologies) according to manufacturer's instructions. Biochemical or immunocytochemical analysis was performed at 48–72 h post-transfection.

### Human primary fibroblasts

Fibroblasts were generated from a 6 mm skin punch biopsy taken under local anesthetic following local ethics board approval and informed consent from the patient. Biopsies were dissected into 1 mm pieces and cultured in 5 cm^2^ petri dishes in media until fibroblasts grew out from the explants. When fibroblasts reached confluency they were transferred to larger culture vessels for expansion. Fibroblasts were generated from a single normal control individual and a PD subject harboring a c.1858G>A, p.Asp620Asn mutation in the *VPS35* gene, as described (33).

### Immunocytochemistry

Primary neurons or fibroblasts were fixed in 4% paraformaldehyde (PFA) and processed for immunocytochemistry as previously described (32). Images were captured with a Zeiss LSM 700 laser scanning confocal microscope (Carl Zeiss AG) with a Plan-Apochromat 63×/1.40 oil objective. Acquired images from single *z*-plane (0.1-μm thick) were subjected to deconvolution with HuygensPro software (Scientific Volume Imaging). Co-localization coefficients (Rcoloc) of deconvolved multichannel images were calculated using NIH ImageJ software as previously described (32).

### Neuronal viability and neurite length assays

Primary cortical neuronal cultures were prepared from P1 Sprague-Dawley rats as previously described (32,34). For assessment of neurite length, cultures were co-transfected at DIV 3 with V5-tagged human VPS35 and GFP plasmids at a 10:1 DNA molar ratio (5 µg total DNA per 35 mm dish) using Lipofectamine 2000 reagent (Invitrogen). Cultures were fixed with 4% PFA at DIV 7 and immunostained with mouse anti-V5 antibody (Invitrogen), and anti-mouse IgG-AlexaFluor-633 antibody (Invitrogen). Images were acquired using an EVOS fluorescence digital microscope (Advanced Microscopy Group). ImageJ software was used to measure the length of the longest neurite from control (GFP-positive) and VPS35-expressing (GFP-/V5-positive) neurons. Sampling was performed randomly with ≥30 neurons measured per condition for each experiment from 4–6 independent cultures/experiments. All measurements were performed by investigators blinded to experimental condition. TUNEL labeling was performed as previously described (32) to assess apoptotic cell death in cortical neurons infected with lentiviral vectors expressing V5-tagged VPS35 or GFP at DIV 3 and fixed with 4% PFA at DIV 14. Cell viability was also assessed following treatment of lentiviral-infected cortical neurons seeded in 96-well plates with cellular toxins for 24 h using the alamarBlue Cell Viability assay (Invitrogen).

### Subcellular fractionation

Cerebral cortex from adult mice (C57Bl/6J) was subjected to subcellular fractionation as previously described (32). The following fractions were obtained: total homogenate (H) and nuclear/whole cell pellet (P1), soluble cytosolic (S1, S2 and S3), heavy membrane (P2), light membrane/microsomal (P3), synaptosomal membrane (LP1) and cytosolic (LS1), synaptic vesicle-enriched membrane (LP2) and cytosolic (LS2).

### Human brain tissue

For immunohistochemical analysis, postmortem human brain tissue was obtained through the brain donation program of the Morris K. Udall Parkinson's Disease Research Center of Excellence and the Alzheimer's Disease Research Center at Johns Hopkins Medical Institutions (JHMI) in compliance with local Institutional Review Board and HIPAA regulations. Formalin-fixed, paraffin-embedded tissue sections (10-µm thick) containing cingulate cortex tissue from two normal control and four PD/DLB brains was obtained as indicated in Table 1 and described previously (34). For biochemical analysis, caudate putamen tissue derived from five normal control and seven PD/DLB subjects was obtained from JHMI (Table 1), and frontal cortex tissue derived from four normal control and eight PD subjects was obtained from the archive at Queen Square Brain Bank (QSBB) as previously reported (35) and indicated in Table 2. Flash-frozen tissues were homogenized with (10% w/v) lysis buffer [20 mM Tris–HCl pH 7.4, 150 mM NaCl, Complete protease inhibitor and phosphatase inhibitor cocktails (Roche Applied Science)] and equal proteins were resolved by 3–8% Tris–acetate sodium dodecyl sulphate-polyacrylamide gel electrophoresis (SDS-PAGE) (Invitrogen). Blots were probed with anti-VPS35, anti-actin or anti-β-tubulin antibodies.

### Recombinant AAV2/6 virus production

V5-tagged human VPS35 (WT or D620N) cDNAs were amplified by PCR from a pLenti6-VPS35 vector and cloned into a pAAV-mPGK-MCS vector (16), modified from the serotype 2 pAAV-CMV-MCS vector (Stratagene, La Jolla, CA, USA). The same backbone plasmid was used to produce the non-expressing control virus. Recombinant AAV2 genome packaged in the AAV serotype six capsid was produced and titrated as described previously (16,36). Viruses were diluted to a final concentration of 2.5 × 10^9^ transducing units (TU) per ml.

### Stereotactic surgeries

Female adult Sprague-Dawley rats (Charles River Laboratories, France) weighing 180–200 g received a viral titer of 5 × 10^6^ TUs (in a 2 µl volume), unilaterally, at coordinates corresponding to the rat substantia nigra, as previously described (36). Animals were sacrificed at 12 weeks postsurgery.

### Biochemical analysis of cell culture and tissues

Cells or brains were homogenized with lysis buffer containing 1% Triton X-100, 50 mM Tris–HCl pH 7.5, 150 mM NaCl, 5% glycerol, 1 mM EDTA, Complete protease inhibitors (Roche Applied Sciences) and phosphatase inhibitor cocktails 2 and 3 (Sigma). Rat tissues for biochemical analysis were harvested at 12 weeks after viral delivery (*n* = 4 animals/group). Ventral midbrains from injected and non-injected hemispheres were rapidly dissected and subjected to mechanical homogenization with (10% w/v) lysis buffer. Cell and tissue lysates were centrifuged at 100 000*g* for 30 min at 4°C. To obtain the insoluble fraction, Triton X-100-insoluble pellets from fibroblasts were solubilized in buffer containing 2% SDS, 50 mM Tris–HCl pH 7.4 and Complete protease inhibitors (Roche). Equivalent proteins as determined by BCA assay (Pierce Biotechnology) were resolved by SDS-PAGE and subjected to western blot analysis with appropriate primary and secondary antibodies. Proteins were visualized by enhanced chemiluminescence (GE Healthcare) and digital images were captured using a chemiluminescent Image Analysis system (LAS-4000, FujiFilm). Acquired images were subjected to densitometric quantitation using NIH ImageJ software. The relative optical density values for each protein band were normalized to the corresponding levels of β-tubulin, actin or PGK proteins to control for protein loading.

### Immunohistochemistry

Animals were deeply anesthetized and transcardially perfused with saline solution followed by 4% PFA in 0.1 M phosphate buffer (pH 7.3). Brains were immersed in 30% sucrose solution before sectioning using a cryostat (Leica, Jung Frigocut 2800N). Immunohistochemistry on rat and human brain tissue with various antibodies was conducted as described previously (34,37). Silver staining was performed using the FD NeuroSilver kit II following the manufacturer's protocol (FD Neurotechnologies, Ellicott City, MD). Images were captured with the light microscopes AX70 and Slide Scanner VS120-L100 (Olympus) or a Zeiss LSM 700 Laser Scanning confocal microscope (Carl Zeiss AG).

### Cylinder test

The forelimb usage of rats was assessed by placing each animal into a transparent cylinder and recording the exploratory activity for 5 min. The contact of either left or right forelimbs with the cylinder wall, while the animals were in a vertical posture, to support body weight or to initiate lateral stepping movements was scored in a manner blinded to experimental group. Forelimb use asymmetry was quantified by calculating the instances when the impaired (contralateral) forelimb was used as a percentage of total number of both limb usages on the cylinder wall.

### Stereological quantitation of neurons

Unbiased stereological estimation of the number of dopaminergic neurons was performed using the optical fractionator probe of the StereoInvestigator software (MicroBrightField Biosciences), as described (37,38). Every fifth serial coronal section of 40 μm thickness, covering the entire substantia nigra region was immunostained with rabbit anti-TH antibody and counterstained with cresyl violet stain. Sampling was performed in a systematic random manner using a grid of 220 × 240 μm squares covering the substantia nigra overlayed on each section and applying an optical dissector consisting of a 75 × 75 × 20 μm square cuboid. All analyses were performed by investigators blinded to each condition.

The optical density of TH-immunoreactive nerve terminals was quantified throughout the striatum as a measure of dopaminergic innervation. The rostral and caudal limits of the striatum used for analysis were 2.00 to −0.50 mm relative to bregma, and every 10th serial section within this region was examined. Following immunolabeling with anti-TH antibody, sections were scanned at 4× magnification using a virtual slide microscope scanning system (VS110, Olympus) and quantified using NIH ImageJ software.

## FUNDING

This work was supported by funding from the Michael J. Fox Foundation for Parkinson′s Research (D.J.M.), the Ecole Polytechnique Fédérale de Lausanne (D.J.M.), the Johns Hopkins University Morris K. Udall Parkinson′s Disease Research Center of Excellence (NINDS P50NS38377; J.C.T.) and the JHU Alzheimer′s Disease Research Center (NIA P50AG05146; J.C.T.). R.B. is funded by the Reta Lila Weston Institute. P.A.L. is a Parkinson's UK research fellow (grant F1002).
